# The Value of T2* in Differentiating Metastatic from Benign Axillary Lymph Nodes in Patients with Breast Cancer - A Preliminary *In Vivo* Study

**DOI:** 10.1371/journal.pone.0084038

**Published:** 2014-01-16

**Authors:** Chuanming Li, Shan Meng, Xinhua Yang, Jian Wang, Jiani Hu

**Affiliations:** 1 Department of Radiology, Southwest Hospital, Third Military Medical University, Chongqing, China; 2 Department of Breast Surgery, Southwest Hospital, Third Military Medical University, Chongqing, China; 3 Department of Radiology, Wayne State University, Detroit, Michigan, United States of America; Rajiv Gandhi Centre for Biotechnology, India

## Abstract

**Background:**

Accurate detection and determination of axillary lymph node metastasis are crucial for the clinical management of patients with breast cancer. Noninvasive imaging methods including ultrasound (US), computed tomography (CT), or conventional magnetic resonance imaging (MRI) are not yet accurate enough. The purpose of this study was to investigate the value of *in vivo* T2* in differentiating metastatic from benign axillary lymph nodes in patients with breast cancer.

**Methodology/Principal Findings:**

In this institutional review board approved study, 35 women with breast cancer underwent multi-echo T2* weighted imaging (T2*WI) of the axillary area on a 3.0 T clinical magnetic resonance (MR) imaging system. T2* values of pathologically proven benign and metastatic axillary lymph nodes were calculated and compared. Receiver operating characteristics (ROC) analysis was conducted to evaluate the diagnostic ability. The areas under the ROC curve (AUCs) and the confidence intervals (CIs) were assessed. In total, 56 metastatic and 65 benign axillary lymph nodes were identified in this study. For metastatic lymph nodes, the average T2* value (55.96±11.87 ms) was significantly longer than that of the benign lymph nodes (26.00±5.51 ms, *P*<0.05). The AUC of T2* in differentiating benign from metastatic lymph nodes was 0.993. The cut-off value of 37.5 milliseconds (ms) gave a sensitivity of 94.6%, a specificity of 98.5%, a positive predictive value of 98.17 and a negative predictive value 95.54.

**Conclusions:**

I*n vivo* T2* can differentiate benign from metastatic axillary lymph nodes in patients with breast cancer. The high sensitivity and specificity as well as the easiness suggest its high potential for use in clinical practice.

## Introduction

Breast cancer is the second leading cause of cancer related mortality in American women with more than 200,000 new cases diagnosed each year [1]. The spread of breast cancer from local lesions to the surrounding lymphatic structures is a well-recognized transformation from the non-metastatic to the metastatic state. It is one of the factors that define prognosis and profoundly alters the required medical and surgical management [2]–[4]. Therefore, accurate detection of axillary nodal metastases is crucial for patients with breast cancer.

Many techniques have been developed to detect axillary lymphatic node metastases. Pathologic analysis requires axillary lymph node dissection or sentinel lymph node biopsy (SLNB), both of which is invasive procedures and can lead to complications of lymphedema [5]. Noninvasive imaging methods including ultrasound (US), computed tomography (CT), or positron emission tomography/CT are not yet accurate enough for clinical practice [6]–[9]. Conventional MRI, dynamic contrast enhanced MRI and diffusion weighted imaging has also proven to be insufficient [10]–[12]. Although MRI with small super paramagnetic iron oxide nanoparticles (SPION) has been documented to assess axillary lymph node status with a high positive predictive value and negative predictive value, there are safety concerns and the clinically available SPION contrast agents were removed from the market recently [13]–[15].

A recent *in vitro* MRI study on dissected axillary lymph nodes in a magnetic field strength of 7 T demonstrated that T2* relaxation time of metastatic nodes was significantly longer than that of benign ones for breast cancer patients [16]. To our knowledge, there is no existing study that use *in vivo* T2* for this purpose. Thus, the aim of this study was to investigate the value of *in vivo* T2* from a 3T clinical MRI system in differentiating metastatic from benign lymphatic nodes for patients with breast cancer.

## Materials and Methods

### Ethics Statement

All research procedures were approved by the Institutional Review Board of the Third Medical Military University and were conducted in accordance with the Declaration of Helsinki. Written informed consent was obtained for all patients.

### Patients Inclusion Criteria

The study population consisted of 35 patients (ages ranging from 30 years to 58 years) with breast cancer, including 27 ductal carcinoma, 7 lobular carcinoma and 1 tubular carcinoma. Patient inclusion criteria were: (1) having clinical T1–2 breast cancers and (2) undergoing SLN or/and axillary lymphadenectomy. Patients who were pregnant, carried an implanted device or had any contraindication for MRI were excluded. Patients who had undergone chemotherapy or radiation therapy within 12 months before the MRI were also excluded from the study.

### Magnetic Resonance Imaging

MRI for all subjects was performed on a 3.0 T whole body system (Siemens Healthcare, Erlangen, Germany) with a 12-channel matrix body coil without intravenous or oral contrast enhancement. Patients were placed in supine position with their arms elevated, head first in the scanner, similar to the position used during surgery and trying to put the axilla near the center of the scanner. Conventional MRI includes transverse T2-weighted images (TR/TE = 4000/70 ms, flip angle = 80°) and T1-weighted images (TR/TE/ = 200/2.78 ms, flip angle = 70°). A multi-echo transverse T2*WI sequence was obtained from the upper thorax to axilla with the following parameters: TE = 4.15, 8.19, 12.23, 16.67, 20.31, 24.35, 28.39, 32.43 ms, TR = 52 ms, flip angle = 15°, number of acquisitions = 1, the total acquisition time was not longer than 3 minute. For all sequences, field of view (FOV) was 200×200 mm^2^ (allowed only one breast to be scanned), the matrix was 256×256, and slice thickness was 3 mm with no gap. The homogeneity of the MRI scanner was checked for each patient.

### Pathology

All patients underwent SLN biopsy with the blue-dye method. After SLNB, the patients underwent mastectomy or breast-conserving surgery, based on the guiding of NCCN 2010. Two physicians with both surgical and radiological licenses were in charge of labeling the lymph node samples. These two reviewers were not involved in the subsequent blinded review and quantitative analysis of the data set. They took part in every MRI examination and in the surgery of every enrolled patient. First, the SLNs were removed at the beginning of the operation. Then, if the patient accepted axillary dissection to level II or to level III, nodes in these two levels were removed. Nodes at the level I were the last to be removed and normally departed from breast sample. After surgery, each node larger than 5 mm was removed from 3 samples of level I to level III were numbered to correspond with that on the MRI image based on their location established with coordinates from clear anatomical landmarks. If several nodes lie close to one another, they were discriminated by size and morphological character [17]. Finally, all dissected sentinel lymph nodes were cut into parallel slices of 2 to 3 mm thickness and embedded in paraffin. Sections were stained with hematoxylin and eosin and histopathologic results were reported as the golden standard in statistical analysis.

### Imaging Analysis

MR images were analyzed by one radiologist (12 years of experience in breast MRI, observer 1) online on a multimodality computer platform (Syngo Multimodality Workplace, Siemens Healthcare, Erlangen, Germany). Inter-observer variability was assessed by a second radiologist (8 years of experience in breast MRI, observer 2), who was blinded to the results of the first observer. Both of them were unaware of the benign or metastasis nature of the lymph nodes and had no access to the other sequences and images. The selection of ROI (Region of interest) was performed manually on every slice of every lymph node, excluding cross sections of fatty center. T2* values were automatically calculated by the commercial workstation. The color T2* map was windowed to provide a visual range of T2* values from low to high, with black representing the lowest T2* value and red representing the highest T2* value.

### Statistical Methods

T2* values of the benign and metastatic lymph nodes were compared using the Generalized estimated equations method. Receiver operating characteristics (ROC) analysis was conducted. The areas under the ROC curve (AUCs) and the confidence intervals (CIs) were assessed. The optimal cut-off value which maximizes the sum of sensitivity and specificity were determined and set as the point in the most upper left hand corner. P<0.05 was considered as statistically significant. All statistical analyses were performed with the SPSS 17.0 software package (SPSS Inc., Chicago, IL, USA).

## Results

Totally 138 nodes were excised and identified by pathologic analysis in this study. No lymph node found on MRI with a T2* fitting with metastasis was not resected. 121 (87.68%) axillary lymph nodes including 56 metastatic and 65 benign nodes were larger than 5 mm and correlated with MR images successfully.

**Figure 1:**
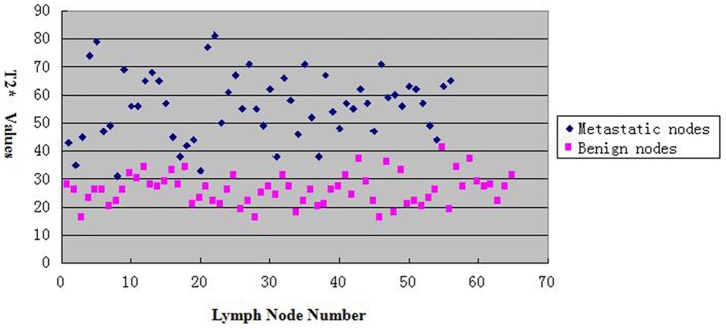
A plot of the T2* values of each metastatic and benign axillary lymph nodes. The horizontal axis indicates the lymph node number, and the vertical axis indicates the T2* values. Black rhombus represents metastatic nodes and red squares represent benign nodes, respectively.

**Figure 2:**
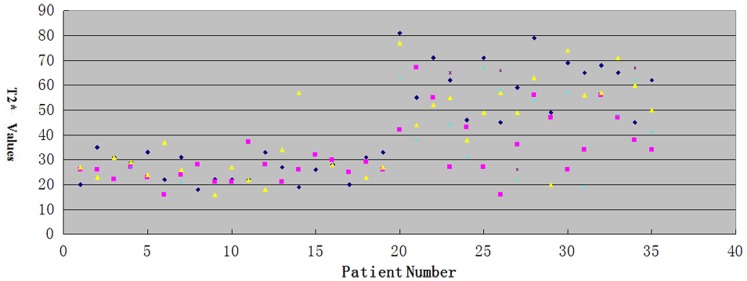
A plot of the T2* values of metastatic and benign axillary lymph nodes for each patient. The horizontal axis indicates the patient number, and the vertical axis indicates the T2* values.

**Figure 3:**
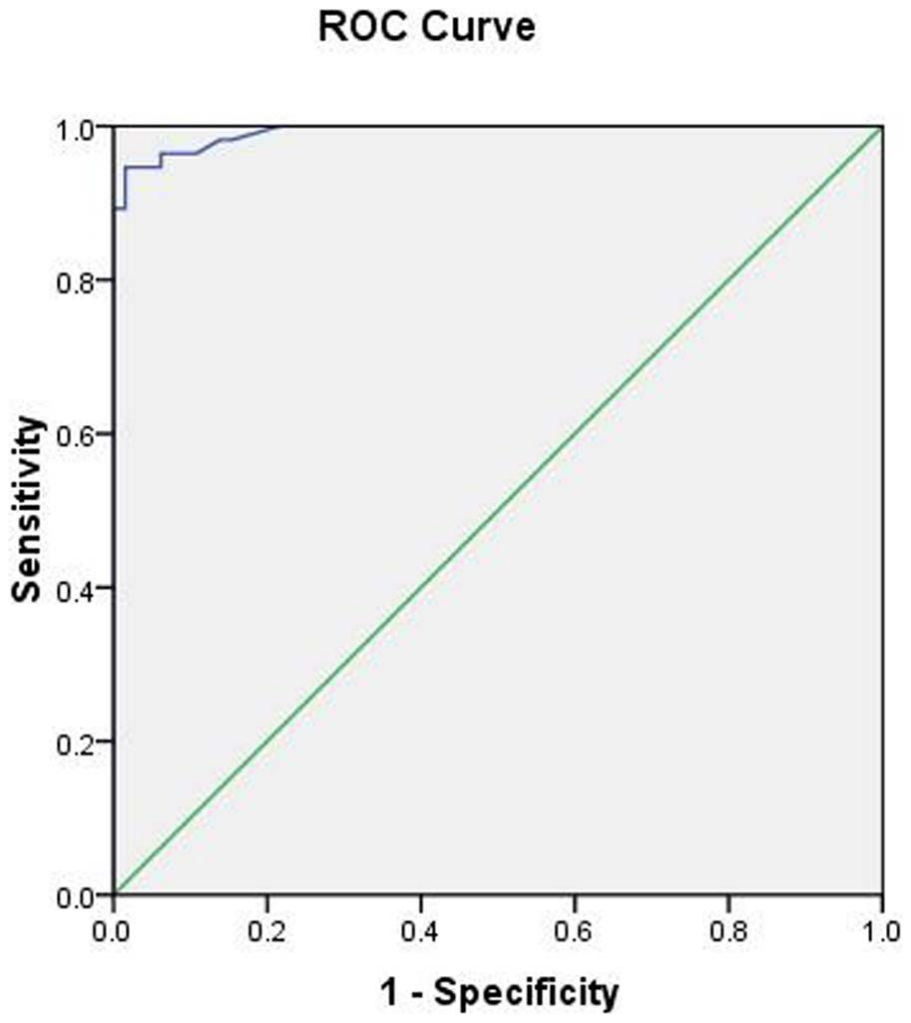
Receiver operating characteristics curves for T2* value in metastatic and benign axillary lymph nodes discrimination.

For metastatic lymph nodes, the average T2* value (55.96±11.87 ms) was significantly longer than that of the benign lymph nodes (26.00±5.51 ms, *P*<0.05) (Figure 1, 2). The AUC for T2* in differentiating benign from metastatic lymph nodes was 0.993. The best cut-off value was 37.5 ms, which gave a sensitivity of 94.6%, a specificity of 98.5%, a positive predictive value of 98.17 and a negative predictive value 95.54 (Figure 3).

No significant difference were found between measurements of the two observers (*P>*0.05).

**Figure 4:**
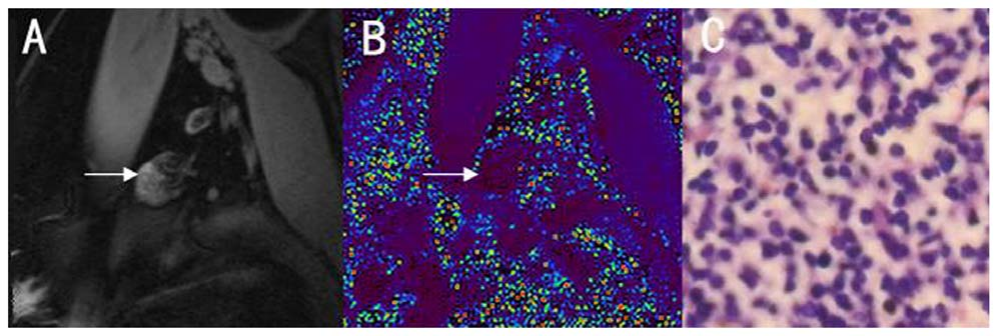
Benign lymph node in a 43-year-old woman. A: T2*WI, B: T2* map, with black representing the lowest T2* value and red representing the highest T2* value. C: corresponding histology image. The benign lymph node displays lower signal intensity than background on the T2* map.

**Figure 5:**
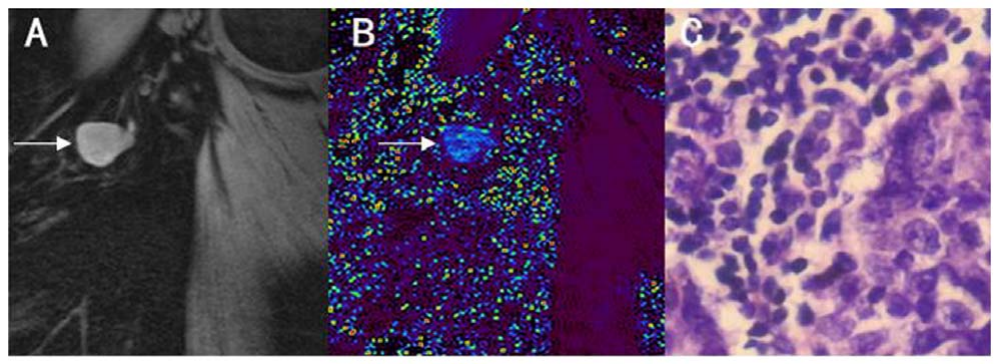
Metastatic lymph node in a 51-year-old woman. A: T2*WI, B: T2* map, with black representing the lowest T2* value and red representing the highest T2* value. C: corresponding histology image. The metastatic lymph node displays higher signal intensity than background on the T2* map.

Figures 4 and 5 showed typical cases of pathologically confirmed benign and metastatic lymph nodes, respectively. Compared to background, the metastatic lymph nodes showed high signal intensity, while benign lymph nodes showed low signal intensity.

## Discussion

The importance of accurately staging axillary lymph nodes for patients with breast cancer is well recognized. Determination of negative lymph node metastasis can help avoid unnecessary axillary dissection, which prevents patients from being exposed to complications including lymphedema, pain, numbness, and limited shoulder movement [18]. Axillary lymph node metastasis has been shown to be an important factor in determining the prognosis for patients with breast cancer. For example, the chance for 5-year survival for patients with node-negative disease is 82.8% compared with 73% for 1–3 positive nodes, 45.7% for 4–12 positive nodes, and 28.4% for ≥13 positive nodes [19]–[21]. The majority of the recommendations concerning axillary radiotherapy are based on the determination of axillary metastatic nodes. Patients with axillary lymph node metastases may benefit from post mastectomy radiation, but the use of post mastectomy radiation in patients with early-stage breast cancer without node metastasis is not supported [4], [22].

Histopathological examination of SLNB is commonly used to detect axillary lymph nodes metastasis. However, SLNB is invasive and about 5 percent of them bear complications of lymphedema [5]. On the other hand, false negative results exist when there is a distant lymph node metastasis with negative SLNB [23], [24]. Enormous effort has been put to develop a noninvasive imaging technique to accurately stage both sentinel and distant axillary lymph nodes for patients with breast cancer. US, CT and conventional MRI techniques mostly focus on morphological criteria, mainly the size of the axillary lymph node. Although larger nodes tend to have a higher incidence of malignancy, benign nodes can be as large as metastatic nodes. It has been well documented that nodal size alone is not an accurate criterion for differentiating metastatic lymph nodes from benign nodes [8]. The dynamic contrast enhancement MRI has been proven to be low in sensitivity for detecting axillary lymph node metastases also [25], [26]. Recently diffusion-weighted magnetic resonance imaging has been intensively investigated. But the results remain controversial [10], [27], [28].

Our preliminary study indicated that the T2* values of metastatic axillary lymph nodes were significantly longer than those of benign nodes for patients with breast cancer. The cut-off value of 37.5 ms gave a high sensitivity of 94.6% and a high specificity of 98.5%. This result suggests that *in vivo* T2* is a promising MRI biomarker for differentiating metastatic from benign lymph nodes. Compared with other reported MRI biomarkers in the literature, such as contours (sensitivity, 35.7%; specificity, 96.7%), cortical thickness (specificity, 63.6%; specificity, 83.2%), short axis length (sensitivity, 83.6%; specificity, 81.2%), long axis length (sensitivity, 78.4%; specificity, 70.9%), shape (sensitivity, 79.1%; specificity, 68.4%), margin (sensitivity, 51.5%; specificity,86.3%), ADC (sensitivity, 88.1%, specificity, 72.6%), dynamic contrast-enhanced MRI (sensitivity, 25.0%; specificity,98.0%) and SPION enhanced MRI (86.4% sensitivity, 97.5% specificity), T2* value is currently the most accurate biomarker in terms of both sensitivity and specificity [9], [28]–[30].

This study was inspired by a previous study on *in vitro* T2* of dissected axillary lymph nodes on a 7T imaging system [14]. Our results confirmed the value of T2* for differentiating metastatic from benign axillary lymph nodes. It is intriguing to note, however, that the difference in T2* between metastatic and benign is smaller for 7T *in vitro* compared to 3T *in vivo*. This is against common expectations for higher versus lower magnetic field as well as *in vitro* versus *in vivo* study. The mechanism behind this anomaly is not known. It is probably due to the difference in the effect of deoxygenated hemoglobin for tissues between *in vitro* and *in vivo* lymph nodes. It is well known that deoxygenated hemoglobin can significantly affect T2* value of tissues [31]. This topic deserves further investigation.

This study had several limitations. The main limitation was all selected patients having breast cancer and undergoing SLN or/and axillary lymphadenectomy, which could introduce a bias. The second limitation is related to the method we used to generate the ROI for evaluation. In our study, we placed ROIs manually and calculated the T2* value over the entire lymph node. This method has the possibility of including cystic or calcified components in our measurements. Lastly, because of the limited MRI resolution, micrometastases and isolated tumor cells were not considered in this study.

In conclusion, we have demonstrated the potential of *in vivo* T2* in differentiating metastatic from benign axillary lymph nodes for patients with breast cancer. The high sensitivity and specificity as well as the easy protocol suggest its high value in clinical practice.
